# Hybrid Navigation Technique for Improved Precision in Implantology

**DOI:** 10.7759/cureus.45440

**Published:** 2023-09-18

**Authors:** Kshitiz Chhabra, Sahana Selvaganesh, Thiyaneswaran Nesappan

**Affiliations:** 1 Implantology, Saveetha Dental College, Chennai, IND

**Keywords:** hand-eye coordination, haptic feedback, static guides, hybrid navigation, dynamic navigation

## Abstract

The hybrid navigation technique involves the merging of the Dynamic navigation (DN) system (Navident, Claronav, Canada) and static navigation technique (3Shape, Copenhagen, Denmark). Combining the advantages of both techniques, devising a protocol of hybrid navigation will be advantageous to eliminate the difficulties faced by operators in using either methods separately. Three patients requiring dental implants were included in this study. This requires the cone beam computed tomography (CBCT) (Digital Imaging and Communications in Medicine (DICOM) data) and intra-oral scan (Standard Tessellation Language (STL) format) data for the accurate planning of the implant positions in both the static and dynamic approaches. The steps carried out were repeated for each of the patients, the accuracy of the implant placement was verified postoperatively by merging the CBCT data pre and post through the Evalunav software (NaviDent, Claronav). The accuracy of the implants placed were assessed based on the mesio-distal, bucco-lingual, apical deviations in distance and in angulation. The semi-robotic DN and static guide combination as a hybrid technique is an interesting method to improve the accuracy of flapless implant surgeries and can be used in cases where the anatomical landmarks are determinant factors for the implant placement.

## Introduction

Dental implants have been an effective treatment option in the management of edentulous arches [1]. Implantologists have multiple options when it comes to planning and placing an implant. Technology has benefited the placement of implants and the capability of office-based imaging and complex stimulation and planning software [2]. Static guides (3Shape, Copenhagen, Denmark) have been a shot in the arm for an implantologist to achieve a predictable accuracy in implant placement [3]. Dynamic navigation (DN, Navident,Claronav, Canada) provides clinicians real-time navigation to improve the accuracy of implant placement [4].

DN is currently used by many medical specialties, including ophthalmology, otolaryngology, neurosurgery, and surgical oncology [5]. Medical DN systems were used for craniomaxillofacial-based procedures. In the 20th century, to assist in the placement of dental implants, additional systems, such as Navident, have been approved and have been providing very reliable results. DN is an advanced surgical technology that uses real-time imaging and computer software to guide surgical instruments and implants during a procedure [5]. The technology offers greater accuracy and precision compared to traditional surgical techniques, which can help improve outcomes for patients. DN works by using a combination of imaging technology and computer software to create a three-dimensional (3D) model of the patient's anatomy. This model is then used to guide the surgeon's instruments and implants in real time during the procedure.

The imaging technology used in DN includes computed tomography (CT) scans. These imaging techniques provide high-resolution images that can be used to create a detailed 3D model of the patient's anatomy. Once the 3D model has been created, the surgeon can use computer software to plan the surgical procedure. The software allows the surgeon to visualize the patient's anatomy and plan the optimal approach to the surgery. The software also allows the surgeon to create a virtual pathway for surgical instruments and implants to follow during the procedure.

During the surgery, the DN system uses sensors and cameras to track the position of the surgical instruments and implants in real time. The DN system works on the basis of triangulation theory where in the stereo-camera, the sensors on the patient's jaw and the sensors on the handpiece with the dominant hand of the surgeon forms the triangle. The system compares the position of the instruments and implants to the 3D model of the patient's anatomy, and provides real-time feedback to the surgeon [6]. This feedback can help the surgeon adjust the position of the instruments and implants as needed to ensure optimal placement and accuracy.

In orthopedic surgeries, the technology can be used to guide the placement of implants, such as hip and knee replacements. In neurosurgeries, the technology can be used to guide the placement of instruments during brain and spinal surgeries. In ear, nose, and throat (ENT) surgeries, the technology can be used to guide the placement of instruments during sinus and skull base surgeries [7,8].

One of the main advantages of DN is its ability to improve surgical accuracy, precision, and patient and operator comfort [9]. The real-time imaging and feedback provided by the system can help surgeons avoid critical structures and ensure that the surgical instruments and implants are placed in the correct position [4,5]. This can help reduce the risk of complications and improve patient outcomes. DN is a powerful surgical technology that offers many benefits for patients and surgeons. As the technology continues to advance, it is expected to become an increasingly important tool in the field of surgery. Static guides and DN are both advanced surgical technologies that can be used to guide the placement of implants during surgical procedures. While these technologies can be used independently, they can also be used together to provide even greater accuracy and precision [5].

Static guides are custom-made devices that are created from a 3D model of the patient's anatomy. The guides are designed to fit over the patient's bone and provide a template for the surgeon to follow during the implant placement procedure. The guides are typically used in conjunction with X-ray imaging to ensure accurate placement of the implant [2].

When static guides are used in conjunction with DN, the result is a hybrid technique that combines the benefits of both technologies. The static guide provides a physical template for the surgeon to follow during the procedure, while the DN system provides real-time feedback and guidance to ensure optimal placement of the implant. During the procedure, the surgeon attaches the static guide to the bone and uses the DN system to guide the placement of the implant. The DN system provides real-time feedback to the surgeon, allowing them to make adjustments to the position of the implant as needed.

The use of static guides with DN can improve the accuracy and precision of implant placement, particularly in complex procedures. The combination of the physical guide and the real-time feedback provided by the DN system can help ensure optimal placement of the implant, which can improve patient outcomes and reduce the risk of complications. Overall, the use of static guides with DN is an advanced surgical technique that offers many benefits for patients and surgeons. As these technologies continue to advance, it is expected that the hybrid technique will become an increasingly important tool in the field of surgery.

## Case presentation

The cases included in this study were treated at the Department of Implantology, Saveetha Dental College, Chennai, India. The patients requiring dental implants were chosen to improve the accuracy of the implants placed to promote healing and better osseointegration. The procedure was approved by the Institutional Ethical Committee of Saveetha Dental College (approval no. IHEC/SDC/PhD/IMP/1904/22/017). The sequence followed for the procedure is explained in detail as follows.

Sequence of procedure

Radiographic Diagnosis

Case selection was done through the initial assessment of cone beam computed tomography (CBCT), and intra-oral impressions were made of the patient’s jaw. Intra-oral scanning evaluation of anatomical structures and available bone width and height was done. The radiographic evaluation was also done using the Navident radiographic software by feeding the CBCT scan to the software [10]. After evaluation of the scans, the planning of the surgical placement of the implant was initiated. Image acquisition includes obtaining 3D files in .dicom format usually and fed to the Navident software for evaluation. The scan must have a surgical site and required radiographic anatomical structures. 

Implant Planning

The radiographic scans were evaluated, and a diagnostic mockup of the available space for prosthetic rehabilitation was done. An intra-oral scan was taken of the oral cavity and fed to the planning software in .stl (Standard Tessellation Language) format. Superimposition of the radiographic scans and the intra-oral scan was done with the Navident software. An intraoral scanner provides a 3D image of the patients' dentition and occlusion. The images were just surface images and lack density/volume. The accuracy is higher in quadrant images, but full-arch images reduce the accuracy of the intra-oral scanner. Merging of the patient's scan and intraoral scanner image was performed while having multiple areas of coordination between the images for better accuracy of the merging. 

Fabrication of Static Guides

Using the 3Shape software, the intraoral images were used to fabricate a static guide. The static guide was then printed using the DIO Probo 3D printer, and after the evaluation of the adaptation of the static guide on the cast, the guide was light-cured. The planned site of the implant was confirmed by placing the static guide on the dental cast and marking implant sites.

Registration and Calibration Procedure

The DN system needs to be fed with the geometry of the patient tracking array relative to fiducials and implant sites. The workflow included the selection of straight or contra-angle handpieces. The calibration occurred 60 to 80 cm from the camera. The hand piece was rotated and calibrated with the camera. The calibration accuracy was verified between the fiducials and drill. 

*Surgical Technique* 

Confirmation of accuracy by performing frequent system checks is always important, followed by confirming the radiographic landmarks on the screen that are exactly correlating. The depth indicators change in color from green to yellow when the drill is 0.5 mm from the targeted depth. During the surgery, the implant size, width, type, and location can be adjusted based on intra-operative factors as required. The static guide was placed in the oral cavity, and calibration of the drill was checked. The drilling sequence was then followed like in any other case, and implant placement was carried out. 

Postoperative Follow-Up

An immediate postoperative CBCT is indispensable in evaluating the placement of implants and comparing with the planned position of implant through the superimposition of pre- and postoperative CBCT. The final calculation of the deviation of the implant from its planned position was made and compared with free-hand surgery.

Case 1

A patient, aged 52, reported to the Implantology Department of Saveetha Dental College, with the chief complaint of missing teeth in her maxillary posterior region. The patient was diagnosed and was having Kennedy Class 3 partially edentulous arch in her upper posteriors. The patient was informed about all the treatment options available. The patient was then subjected to a radiographic diagnosis, the width and height of the bone in the edentulous site were assessed with the CBCT, and the results show that the bone in the particular region followed sub-antral (SA) class 2. Hence, the size of bone available apically under the sinus is about 8-10 mm, requiring indirect sinus lift and simultaneous implant placement in relation to 16 and 17.

**Figure 1:**
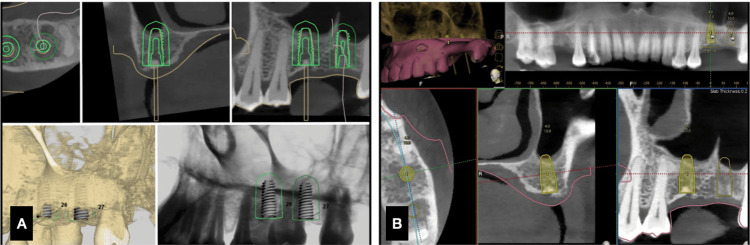
Case 1. A) Adjacent implants planning under 3Shape. B) Implant planning under the Navident software.

**Figure 2:**
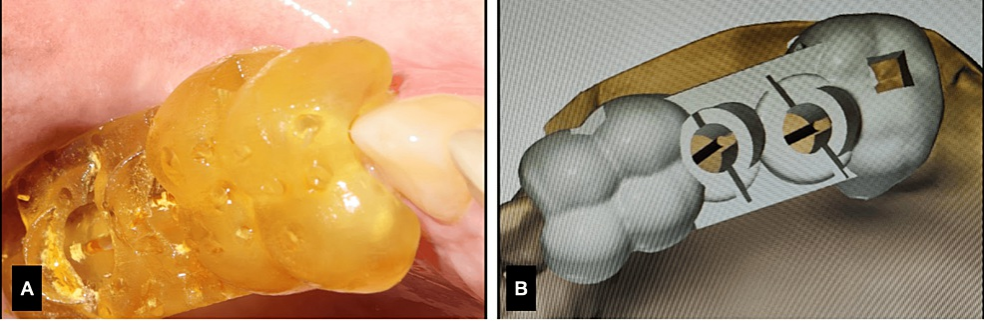
Case 1. A) Surgical guide design, B) surgical guide seating verification.

As the site is the most posterior region and the site warranting crestal approach sinus lifting, the range of mouth opening of the patient was assessed clinically. The preparatory diagnostic impressions and intra-oral scanning were all carried out. The planning of the implant locations under the DN software (Navident) was done (Figure 1). The STL files from this were transferred to the static guide planning and fabrication software (3Shape) (Figure 2). The position of the implants were aligned with that of the DN planning. The surgical guide was printed and checked for the accuracy of fitting in the cast.

**Figure 3:**
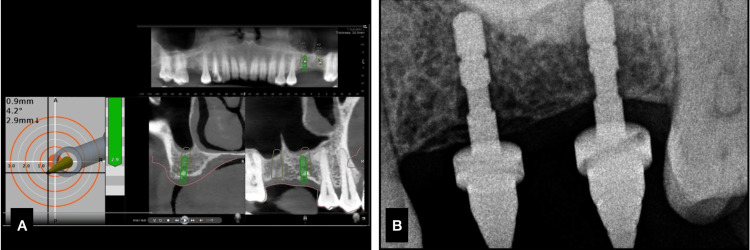
Case 1. A) Drilling under Dynamic navigation, B. position indicating device (PID) radiograph.

**Figure 4:**
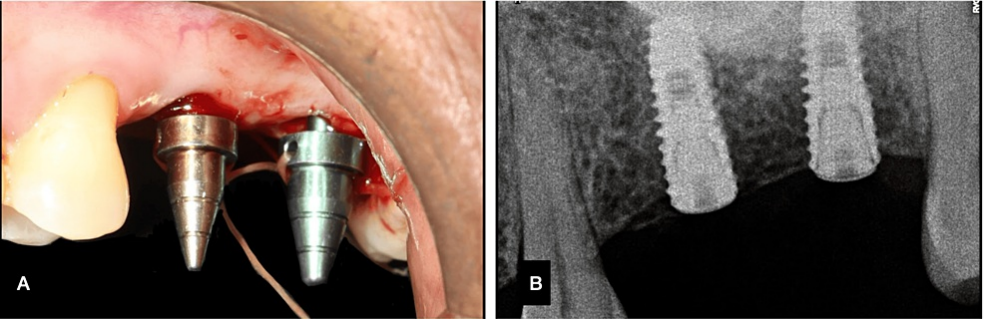
Case 1. A) Clinical PID verification, B) implant placement radiograph.

On the day of surgery, the Crestal Approach Sinus KIT (CAS-KIT), with a guided drill kit (Osstem, UK), was used. The guided drill kit was used till the 2 mm drill to ensure the ultimate mesio-distal and bucco-lingual precision of the implant placement. Post the 2 mm drill, sequential drilling was followed with the DN plan. This ensures that the implant placement is guided in real time (Figures 3,4). The sinus lift was carried out under DN, with the help of two bookmark options available in the software (two treatment plans: one till the sinus wall and the other involving the sinus). A Bio-Oss graft (Geistlich, Switzerland) was used for the indirect sinus lift. Osstem implants with a dimension of 4 x 10 mm were placed in relation to 16 and 17. The advantage of treating this case particularly under the hybrid navigation technique is that the surgical site was kept clean and the flapless approach was maintained even if it was an indirect sinus lift case, which required pronounced accuracy in sinus lift and implant placement.

**Figure 5:**
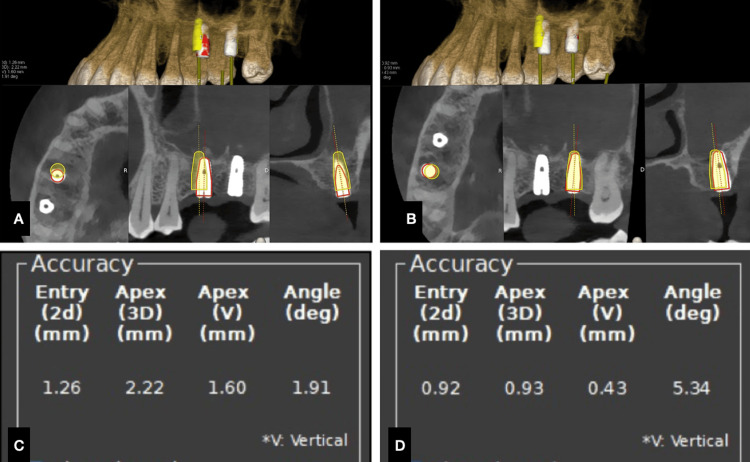
Case 1. A) Evalunav analysis of the first implant, B) Evalunav analysis of the second implant, C and D) accuracy values for the implants placed.

Post implant placement, the patient was re-subjected to CBCT to assess the accuracy of implant placement with that of the implant planning. Evalunav (Navident, Claronav) software was used to merge the plan and the post implant placement. The entry (mm), apex (3D), apex (vertical), and angle (degree) deviations were assessed (Figure 5, Table 1). The values of the same are tabulated below.

**Table 1:** Evalunav values of implants placed in relation to 16 and 17 (Case 1).

Placement parameters	Evalunav deviation values (16)	Evalunav deviation values(17)
Entry (mm)	1.26	0.92
Apex (3D) (mm)	2.22	0.93
Apex (vertical) (mm)	1.60	0.43
Angulation (degree)	1.91	5.34

Case 2

**Figure 6:**
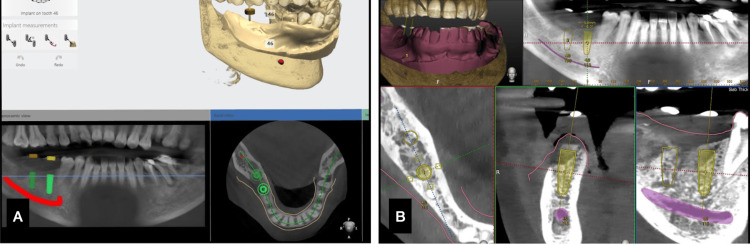
Case 2. A) Adjacent implants planning under 3Shape, B) implant planning under the Navident software.

**Figure 7:**
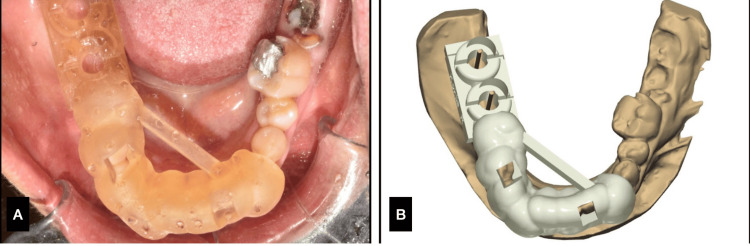
Case 2. A) Surgical guide design, B) surgical guide seating verification.

A female patient, aged 47, reported to the Department of Implantology, Saveetha Dental College, with the chief complaint of missing teeth in her lower posterior region. Treatment options of cast partial denture and dental implants were given to the patient as this case was Kennedy Class 2 involving the lower posteriors. The patient was subjected to radiographic diagnostics, and impressions were taken. Adequate bone was found in the edentulous site, which warranted dental implant placement. The initial planning was carried out in the DN software. The vital structure and the inferior alveolar nerve were marked, and the implants were planned with respect to the edentulous site. The planning STL was exported to the 3Shape software for the planning of implants and fabrication of the surgical guide (Figures 6,7). 

**Figure 8:**
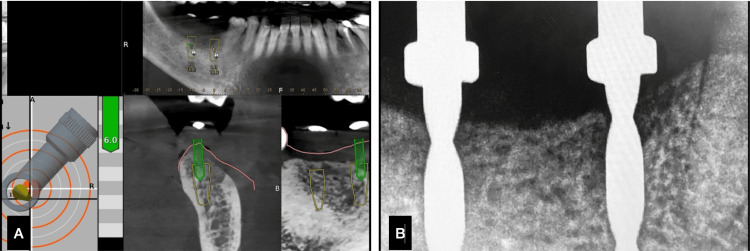
Case 2. A) Drilling under Dynamic navigation, B. position indicating device (PID) radiograph.

On the day of implant surgery, local anesthesia (LA) was given, and the jaw trackers (Y-shaped) were stabilized in position. The merging of the patient data and the clinical alignment happened with the tracers. Teeth adjacent to the site were taken as reference points, the tracer was calibrated, and the trace was registered on the chosen teeth (Figure 8). The accuracy of the trace registration was checked by placing the tracer tips on the tooth surface, and the DN software gave the accuracy of deviation (mm) from the surface. A value of -1 till 1 mm of deviation is accepted.

**Figure 9:**
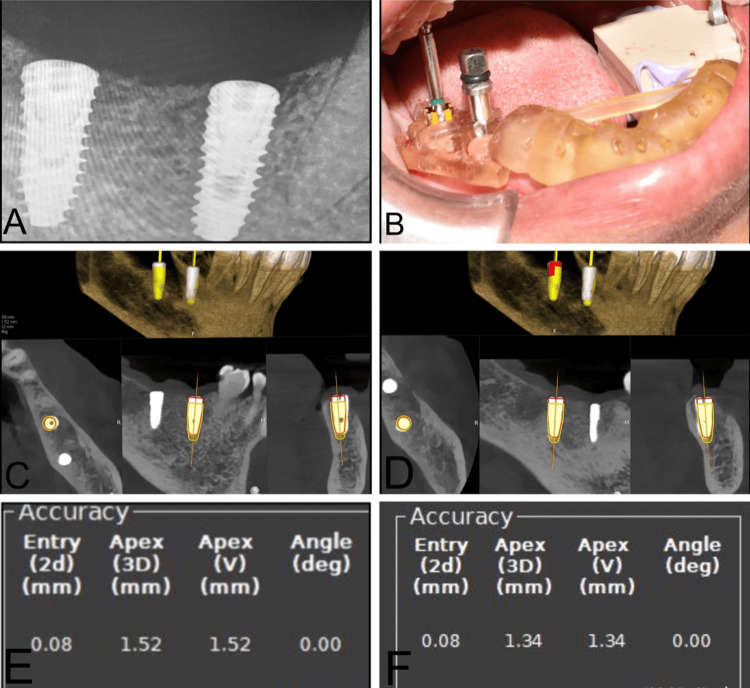
Case 2. A) Clinical position indicating device (PID) verification, B) implant placement radiograph, C) Evalunav analysis of the first implant, D) Evalunav analysis of the second implant, E and F) accuracy values for the implants placed.

Once the trace registration was done and the patient was also comfortable, the surgical guide was put in place, and sequential drilling was done with the calibration of each drill for the specific depth detection. Until the 2 mm drill, the accuracy was maintained by both the static guide and DN. Then, sequential drilling was done by free hand. The implant placement was done through the surgical guide in place. Implants measuring 4 x 10 mm (Osstem) were placed with a primary stability of 30 Ncm. The accuracy and parallelism of the implants were assessed using the Evalunav software, which merges the planning and post-placement CBCT (Figure 9, Table 2). The accuracy of the implant placement is tabulated below.

**Table 2:** Evalunav values of implants placed in relation to 46 and 47 CASE 2

Placement parameters	Evalunav deviation values (46)	Evalunav deviation values (47)
Entry (mm)	0.08	0.08
Apex (3D) (mm)	1.52	1.34
Apex (vertical) (mm)	1.52	1.34
Angulation (degree)	0.00	0.00

Case 3

A patient, aged 24, male, reported to the Department of Implantology with the chief complaint of missing teeth in his upper front tooth region. As the patient was young and as he insisted on esthetics, the treatment plan of implants were suggested to the patient. The patient was subjected to CBCT, impressions and intra-oral scans were taken, and the bone dimensions were assessed. The site was previously grafted due to the friable bone in the site, and the hybrid navigation technique was finalized. The implant planning was done under the DN software, and the same steps were followed like the previous two cases for the fabrication of the surgical guide.

**Figure 10:**
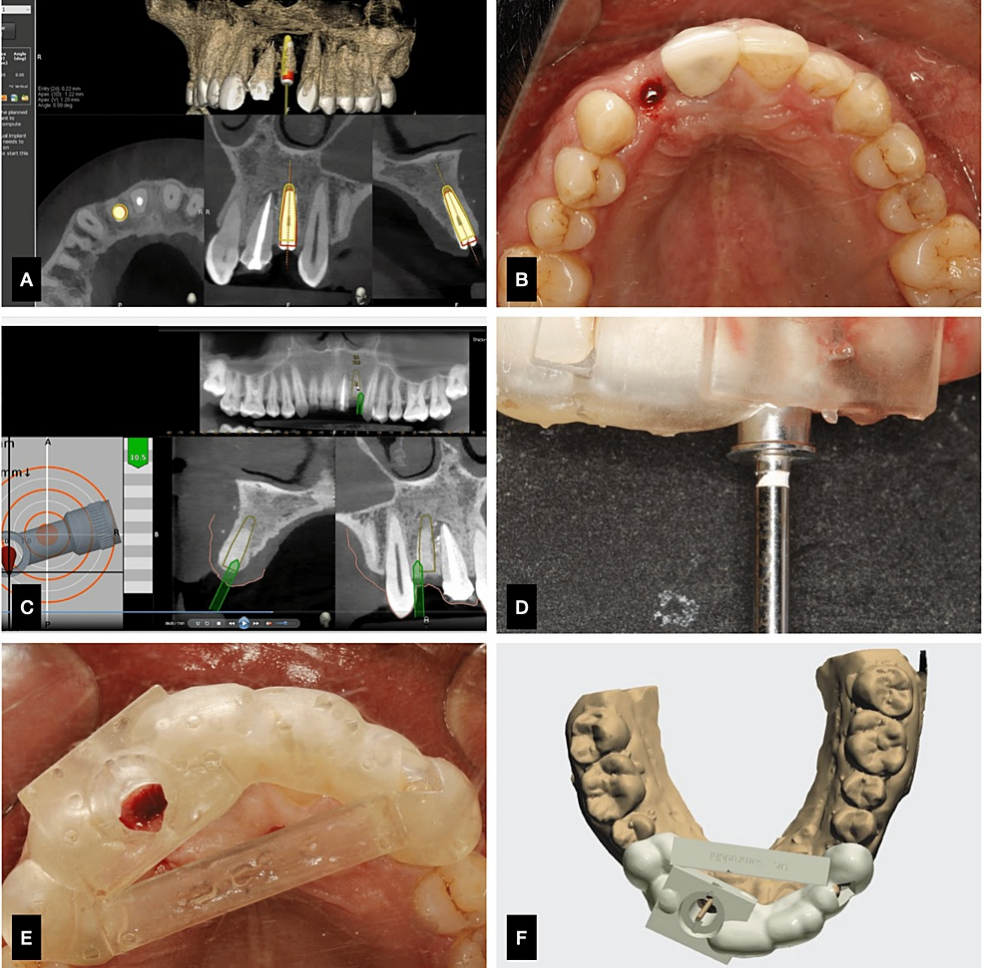
Case 3. A) Evalunav consideration, B) arch with implant placement, C) drilling sequence under DN, D) static guide in place, E) static guide design.

The implant of dimension measuring 3.5 x 10 mm (Osstem) was planned, and expansion screws were used with a handpiece and was calibrated each time for each increasing width. The implant placement was done by reaching a primary stability of 30 Ncm (Figure 10, Table 3). The surgical site in the anterior region was kept clean, and the grafted site was intact, with adequate blood supply. The post-placement accuracy was carried out using Evalunav, and the findings are tabulated below.

**Table 3:** Case 3. Evalunav values of implant placement in relation to 22

Placement parameters	Evalunav deviation values (22)
Entry (mm)	0.22
Apex (3D) (mm)	1.22
Apex (vertical) (mm)	1.20
Angulation (degree)	0.00

## Discussion

The advent of DN has been a boon to dentistry especially implant dentistry. DN is effective in cases where the anatomical structures are vital and the residual bone for implant placement is compromised. Prior to the introduction of the DN system for implantology, there was the usage of static guides, in which the implant's positions can be prosthetically planned and the position of the implant is guided with a stent in place intra-orally [11]. It is of utmost importance in implantology that the angulation of the implants are maintained for the improved marginal fit of the prosthesis and survival of the implant without crystal bone loss for a prolonged period of time [12-19].

The accuracy of the implant placement with the combination of static and dynamic systems is as close to digital planning. Sun et al. found the least deviation with combining the static and DN approaches [20]. The DN system has some drawbacks; as the DN is semi-robotic in nature, there is no robotic arm that is attached to the main machine, and the 3D orientation of the handpiece and the drill is left to the surgeon's skill. There is a deep learning curve associated with that of the DN system. According to Wang et al., the accuracy of implant placement in this in-vitro study was higher with the active DN group than that with the passive DN group [21]. The active DN has infrared cameras detecting light-emitting diodes that are connected with the instruments.

DN requires surgeons to be trained prior to their attempt on patients with the DN unit. The most common reported drawbacks are the heaviness of the hand piece and the hand-eye coordination, which is a difficult feat to achieve without a continued practice with the DN unit. The other challenge associated with the DN is the expert skills of the operating surgeon on the haptic feedback that is an important factor to consider for implants placed under the DN system [22]. The operating surgeon should be able to do the entire procedure without the necessity to look into the patient's mouth, rather to look at the screen during the drilling and placement of the implant.

These shortcomings can be addressed with the use of a hand piece that can be restricted in all directions. The orientation of the hand piece associated with the DN unit and the use of a static guide for the precision and 2 mm drills can be very helpful in providing a path to be followed for sequential drilling and implant placement. This also considerably improve the precision of implant placement. For both to work simultaneously, a proper planning protocol needs to be incorporated [23]. The accuracy of the seating of the surgical guide has to be meticulously checked prior to the start of the surgical procedure. 

Even though the surgical guide offers a high level of accuracy, the errors in the printing of surgical guides and the shrinkage of resins can be a reason for the errors in the seating of the surgical guides. As the procedure is the most important, it is a blinded one, and there is no room for the visualization of the bone or the soft tissues. The seating of the surgical guides are very important as it commands the correct position of implant placement as planned in the software [24]. These errors can be minimized with the usage of DN, which can correct the changes in the guide seating or adjust any hampering in the drilling procedures. 

A factor that should be considered is the lateral tolerance between the metal sleeve of the guide and the surgical drills or the implant transfer. This tolerance should be small enough to improve the accuracy and should not be too tight so that the friction would impede with the placement of the implant. This study used a sleeveless system of Osstem implants, in which the drill kit allows for this tolerance with drills especially designed for this purpose. There is a possibility that the drills are horizontally and angularly displaced even when drilled with the surgical guide in place. During such situations, the feedback from the DN can be critical, as it allows the surgeon to immediately correct the angular or horizontal or vertical deviations [25-27]. Another advantage of using the DN and static guides together is that immediate loading of the implants after placement is also a viable option. If the guides are used until the implant placement is proper, selection of the abutment and crowns can be kept ready, and immediate loading of the implants can also be done. 

As this is a case series, more patient data and improved follow-up can be carried out for an improved understanding of the concept and to throw more light on the accuracy that is obtained.

## Conclusions

The use of DN and static guides yields a positive result. It also improves surgeons' comfort as it restricts the hand piece. The orientation with the drill tag of the hand piece also is improved considerably. The combination of both systems can be carried out to yield more appropriate results. The drawback is that the cost of static and DN units will be drastically high. This is one of the factors that need to be addressed in the near future. There is a need to have a clinical trial with an improved sample size to determine the long-term success and survival of dental implants placed under this method.
